# A Case Series of Lymphangioleiomyomatosis, a Rare Cystic Lung Disease

**DOI:** 10.1002/rcr2.70535

**Published:** 2026-03-10

**Authors:** Sümeyye Sedef, Pınar Mutlu

**Affiliations:** ^1^ Department of Chest Diseasese Çanakkale Onsekiz Mart University Çanakkale Turkey

**Keywords:** pneumothorax, pulmonary lymphangioleiomyomatosis, sirolimus, tuberous sclerosis

## Abstract

Lymphangioleiomyomatosis (LAM) is a rare interstitial lung disease that predominantly affects women of childbearing age. In this case series, we presented five cases diagnosed with LAM in our clinic in the last 2 years. All cases were female, and their ages ranged from 40 to 54 years. The primary symptoms of LAM seen in the cases were exertional dyspnea and cough. Radiological examination revealed multiple bilateral thin‐walled air cysts on high‐resolution computed tomography (HRCT) in all cases. In three cases, the diagnosis of LAM was made histopathologically by wedge resection. Awareness of this rare disease's clinical and radiological features is critical for early diagnosis and timely initiation of treatment.

## Graphical abstract

We studied five women with a rare lung condition called LAM that typically affects only 1–5 women per 10 million. All patients had breathing difficulties and characteristic lung scans showing multiple thin‐walled air sacs. Finding five cases at one center in just 2 years suggests this condition might be more common than previously thought or often goes undiagnosed. Earlier recognition by doctors could help patients receive appropriate treatment sooner, potentially improving their quality of life.

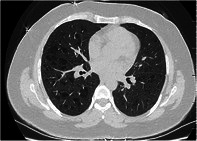

## 1 Introduction

Lymphangioleiomyomatosis (LAM) is a rare idiopathic, diffuse, progressive interstitial lung disease that predominantly affects women of childbearing age. It may develop sporadically or as a component of tuberous sclerosis syndrome. Although the pathogenesis of LAM is not fully known, the fact that it is not seen before menarche and is rarely encountered after menopause [1] suggests that oestrogen has a major role in its progression. LAM patients most commonly present with exertional dyspnea and pneumothorax. The imaging method most widely used in diagnosing LAM is high‐resolution computed tomography (HRCT). HRCT scans of LAM patients typically show diffuse, thin‐walled, round air cysts [2] in both lungs that may tend to coalesce. According to ATS/JRS 2017 clinical practice guidelines, LAM can be diagnosed without biopsy when HRCT is typical and a confirmatory feature is present (e.g., tuberous sclerosis complex, renal angiomyolipoma, chylous effusion or serum VEGF‐D ≥ 800 pg/mL). In our series, in patients without biopsy, diagnosis was established based on typical HRCT features. In the last 2 years, five cases have been diagnosed with LAM in our clinic. In this context, we conducted this case study in order to contribute to the literature by presenting these five cases in the light of the existing literature data. Pulmonary function test (PFT) results, pathology findings and fingertip oxygen saturation were reviewed, but we couldn't find the data for six‐minute walk test (6MWT).

## 2 Case Series

### 2.1 Case‐1

**FIGURE 1:**
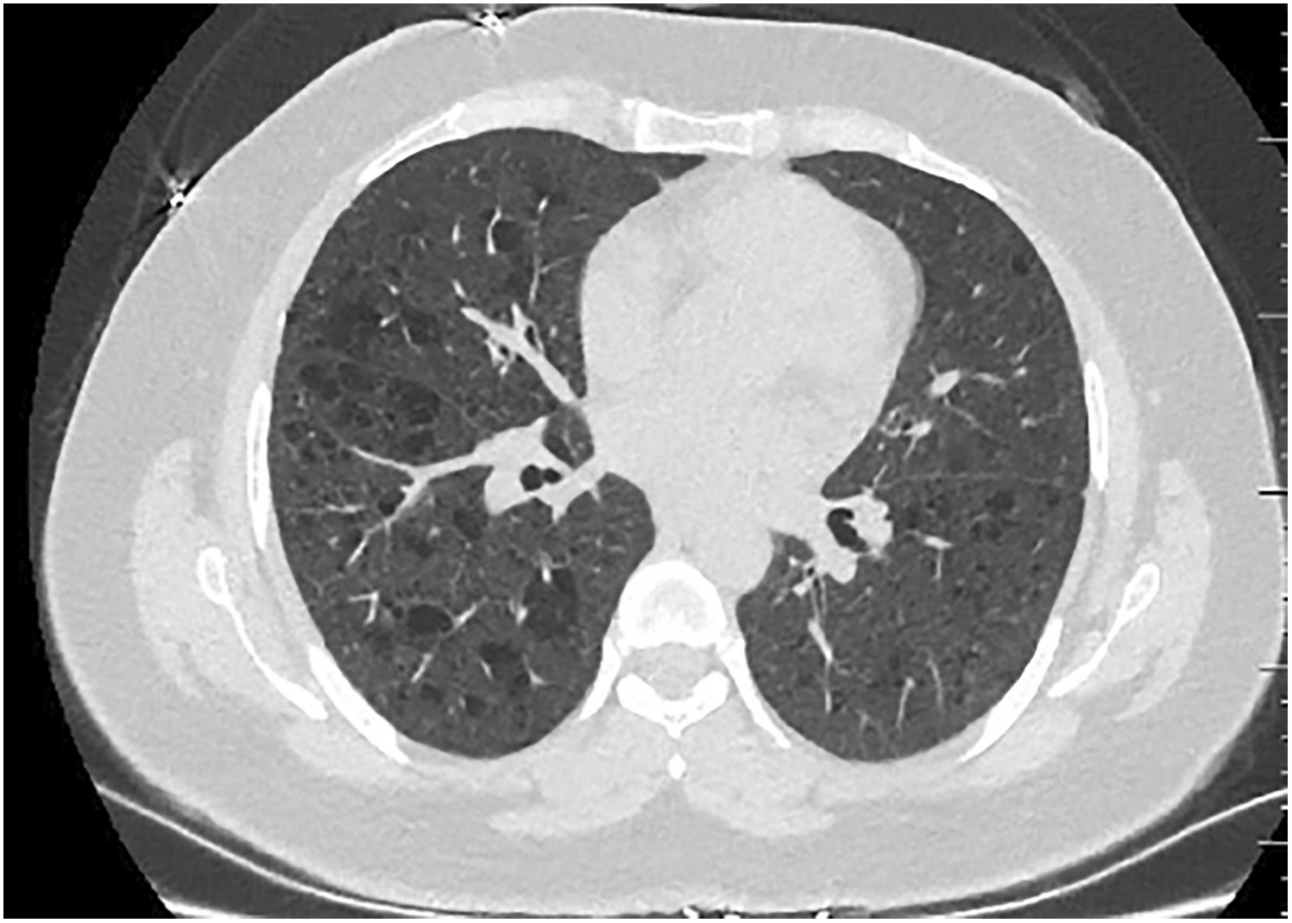
Cross‐sectional thoracic CT image of Case 1 (Image of thin‐walled air cysts and bleb).

A 40‐year‐old female patient presented to another facility with complaints of exertional dyspnea and dry cough, which had recently become worse. She was referred to our clinic based on thoracic CT findings indicating interstitial lung disease. The patient was an active smoker with a five‐pack/year smoking history as well as a history of tuberculosis dating 26 years back. The patient, who had previously worked in a dairy farm, had no known comorbidities. The results of her PFT were found to be restrictive with FVC 2.96 L (93% predicted), FEV_1_ 2.13 L (77% predicted) and FEV_1_/FVC 72%. The carbon monoxide diffusion test revealed her diffusion capacity as 63%. The patient was evaluated for LAM after thoracic CT, which revealed thin‐walled air cysts and bleb formations (Figure 1). She underwent wedge resection by the thoracic surgery department in order to establish a pathological diagnosis. The pathology tests indicated multiple benign lymphatic channels, irregular emphysema, atelectatic changes and desquamation findings in the patient's lung parenchyma. The findings were compatible with the diagnosis of pulmonary LAM. The patient was subsequently started on sirolimus treatment at a dose of 2 mg/day. Symptoms improved and FEV_1_ increased by 820 mL at 1 year follow up.

### 2.2 Case‐2

**FIGURE 2:**
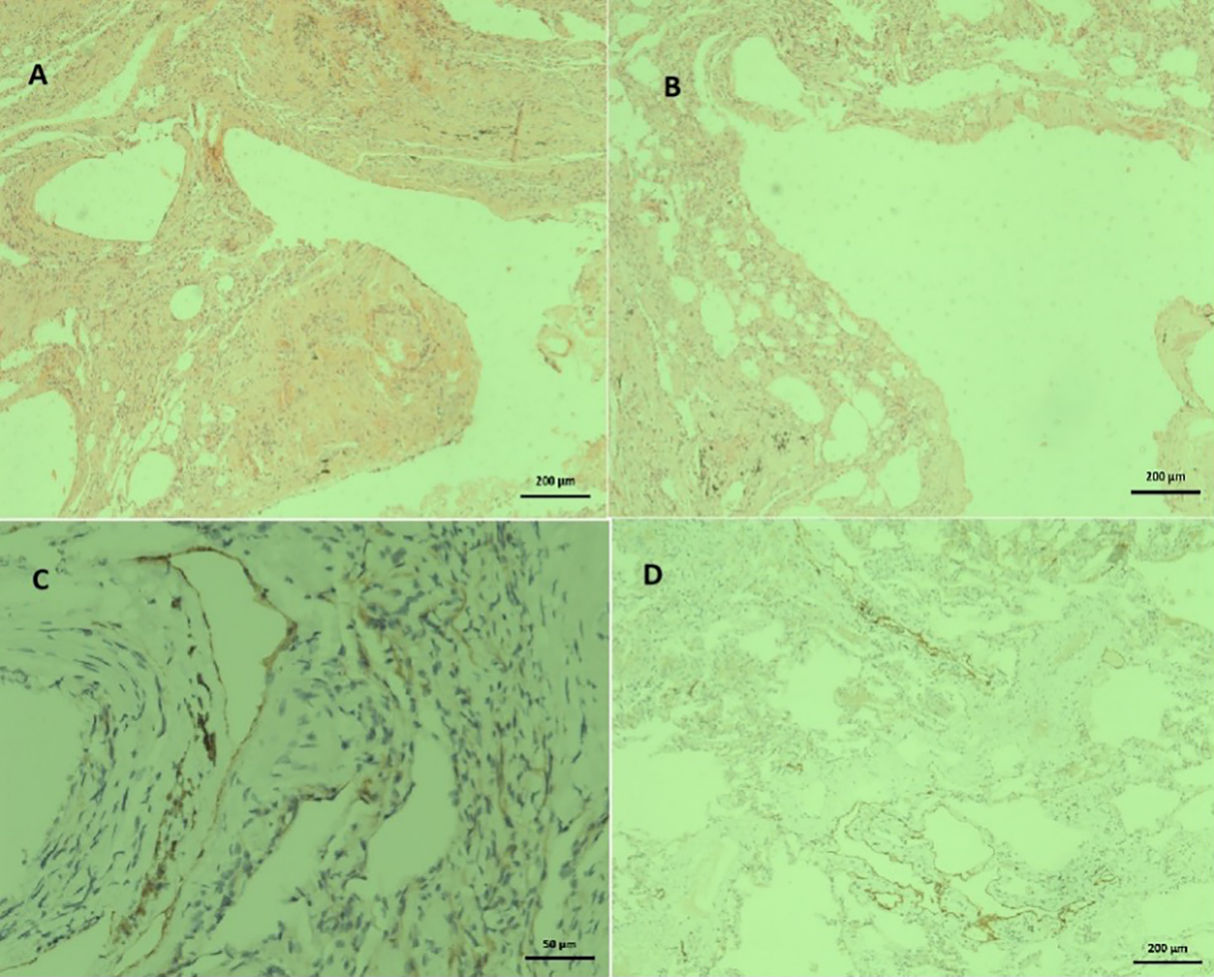
Cross‐sectional pathology images of Case 2. (A, B) Dilated lymphatic channels H&E ×40. (C) Podoplanin dilated lymphatic channels with immunohistochemical antibody staining ×50 mm. (D) Podoplanin dilated lymphatic channels with immunohistochemical antibody staining ×40.

A 42‐year‐old female patient presented to our clinic with exertional dyspnea that had been ongoing for approximately 1 year and exacerbated in the last 3 months. Pulmonary LAM was suspected in the patient due to her examination findings and the presence of multiple air cysts with smooth contours and peribronchial wall thickening in the thoracic CT scan. The patient was scheduled to undergo wedge resection by the thoracic surgery department. Figure 2 contains the pathology photographs of the case. The patient's preoperative PFT were: FEV_1_ 1.66 L (63% predicted), FVC 3.36 L (109% predicted), FEV_1_/FVC 49%, DLCO 47%. Accordingly, inhaler therapy was given to the patient. Following regular inhaler use, the patient underwent wedge resection. The patient was diagnosed with LAM pathologically. Sirolimus 2 mg/day was initiated; dyspnea resolved and FEV_1_ improved by 890 mL at 1 year follow up.

### 2.3 Case‐3

A 54‐year‐old female patient was pathologically diagnosed with LAM in 2011 but did not use medication regularly. The patient applied to our clinic to receive medication with the complaint of increasing shortness of breath. Diffuse thin‐walled air cysts were observed on her thoracic CT scan. The findings were found to be compatible with the diagnosis of pulmonary LAM. The patient's PFT were: FEV_1_ 1.18 L (52% predicted), FVC 2.37 L (89% predicted), FEV_1_/FVC 50%, DLCO 90%. The patient was subsequently started on 2 mg/day sirolimus treatment and followed up. Follow up showed symptom regression and a 16% relative improvement in FEV_1_.

### 2.4 Case‐4

A 52‐year‐old female patient diagnosed with LAM applied to us for a follow‐up on her disease. The patient, who had intermittent shortness of breath and cough, had a history of two right side pneumothorax twice in 2019 treated with tube thoracostomy. Her thoracic CT scan revealed multiple round‐shaped, thin‐walled air cysts, the largest of which measured approximately 18 mm, scattered throughout all lobes in both lung parenchymas. Her findings were found to be compatible with LAM. The patient's PFT were: FEV_1_ 3.16 L (107% predicted), FVC 4.30 L (124% predicted), FEV_1_/FVC 73%, DLCO 70%. The patient was recommended to continue sirolimus treatment at a dose of 1 mg/day. Pleurodesis was not considered in this patient because lung transplantation may be an option in patients with LAM, and pleurodesis can be a contraindication for transplantation. Sirolimus treatment at a dose of 1 mg/day was recommended in order to achieve target trough levels while minimising adverse effects; however, the patient did not consent to the proposed treatment.

### 2.5 Case‐5

Our fifth case was a 48‐year‐old female patient for whom we were consulted for preoperative assessment. The patient, who described intermittent exertional dyspnea without active pulmonary complaints, had no known lung disease. She had a 20 pack‐year smoking history. The PFT revealed a FVC: 4.28 L 128%, FEV1: 2.79 L 98%, FEV1/FVC: 65% and an obstructive pattern. Thoracic CT revealed diffuse air cysts and occasionally accompanying bronchiectasis. The patient was pre‐diagnosed with LAM based on radiological findings, but she did not accept further examination and treatment. LAM was suspected radiologically; genetic testing and biopsy were planned, but the patient declined further diagnostic evaluation and treatment.

## 3 Discussion

LAM affects the lungs, kidneys and the lymphatic system. Whilst clinical stability may be expected in many patients established on sirolimus, disease progression may still occur, warranting consideration of lung transplant referral [1]. Patients with LAM most commonly present with symptoms such as shortness of breath, recurrent pneumothorax, chylothorax and haemoptysis [3]. HRCT scans of patients with LAM typically show an involvement pattern characterised by numerous diffuse bilateral, uniform, round, thin‐walled cysts [2].

The primary goals of LAM treatment are symptom control, improved quality of life and slowing disease progression. Supportive treatments such as oxygen therapy are recommended for those with hypoxemia. Respiratory rehabilitation may be helpful in cases with reduced functional capacity.

Despite the expression of oestrogen and progesterone receptors on LAM cells, the evidence does not support routine introduction of hormonal therapy due to lack of clear efficacy and potential side effects. Transplantation may be more challenging in patients who have previously undergone pleurodesis for pneumothorax or surgical management of recurrent chylothorax. Management of refractory chylothorax may also include a diet low in long‐chain triglycerides.

In this five‐patient LAM case series, typical HRCT patterns, attention to confirmatory features and early initiation of sirolimus were central to management. Our experience underscores practical points: consider mTOR inhibition when lung function declines or chylous complications occur; anticipate pneumothorax and chylothorax and manage proactively (including dietary measures for refractory chylothorax); and refer appropriate candidates for lung‐transplant assessment, particularly when progression occurs despite therapy. These lessons may facilitate earlier recognition and more consistent care pathways.

In this study, we presented five female cases diagnosed with LAM in our clinic over the last 2 years. These cases, ranging in age from 40 to 54 years, reflect the typical demographic characteristics seen in women of reproductive age, consistent with the literature. Exertional dyspnea and cough were prominent symptoms in all five cases, and bilateral thin‐walled air cysts were present on HRCT. Histopathological diagnosis was made by wedge resection in three cases, while the other two cases were diagnosed based on radiological findings and clinical presentation.

Sirolimus treatment was initiated in all our cases and regular follow‐up was recommended. The diagnosis of five LAM cases in our clinic over a 2‐year period highlights the importance of increased awareness of this rare disease and advancements in imaging techniques. This case series demonstrates that knowledge of LAM's clinical and radiological features is critical for early diagnosis and timely initiation of treatment.

Although the global prevalence is not precisely known, it has been reported to occur in 1 to 5 out of 10,000,000 women [4], and the five cases diagnosed in our clinic in a short period suggest that there may be regional differences or that the actual prevalence of the disease may be higher than previously thought. Considering the challenges in diagnosing and treating LAM, a multidisciplinary approach and personalised treatment strategies are necessary.

In the aetiology of LAM, vascular endothelial growth factor‐D (VEGF‐D) is a key factor secreted by LAM cells that promotes lymphangiogenesis, angiogenesis and disease progression via VEGFR‐3. Serum VEGF‐D levels are also used as a clinically valuable biomarker in the diagnosis of LAM [5].

Our cases were diagnosed in accordance with the HRCT and clinical criteria defined in the 2010 European Respiratory Society (ERS) guidelines for LAM. These guidelines recommend the integrated evaluation of characteristic HRCT findings together with clinical and histopathological data, thereby helping to avoid unnecessary invasive procedures [6].

In our case series, diagnostic and therapeutic decisions were made in accordance with the clinical practice guidelines published by the American Thoracic Society and the Japanese Respiratory Society (ATS/JRS). In line with these guidelines, characteristic HRCT findings were considered one of the key adjunctive tools supporting the diagnosis. Furthermore, the ATS/JRS guidelines recommend considering mTOR inhibitors, such as sirolimus, as an effective and appropriate treatment option in selected patients with evidence of disease progression [7].

## Author Contributions

**Sümeyye Sedef:** conceptualisation, data curation, formal analysis, funding acquisition, investigation, methodology, project administration, resources, software, supervision, validation, visualisation, writing – original draft, writing – review and editing. **Pınar Mutlu:** conceptualisation, data curation, formal analysis, funding acquisition, investigation, methodology, project administration, resources, software, supervision, validation, visualisation, writing – original draft, writing – review and editing.

## Funding

The authors have nothing to report.

## Consent

Written informed consent was obtained from all patients using an institutional consent form. The consent form complies with the requirements outlined in the journal's author guidelines.

## Conflicts of Interest

The authors declare no conflicts of interest.

## Data Availability

The data that support the findings of this study are available from the corresponding author upon reasonable request.
